# Carotid Atherosclerosis in Ischemic Cerebrovascular Patients

**DOI:** 10.4021/jocmr2009.03.1226

**Published:** 2009-03-24

**Authors:** Ai Juan Zhang, Ai Yuan Zhang, Chi Zhong

**Affiliations:** a Department of Neurology, Weifang People's Hospital affiliated to Weifang Medical Colllege, Shandong, China, 261041; b Department of cardiology, Weifang People's Hospital affiliated to Weifang Medical Colllege, Shandong, China, 261041

## Abstract

**Background:**

Cerebral emboli resulting from atherosclerosis at the carotid bifurcation is a major cause of ischemic stroke. A convenient and prompt evaluation is necessary for secondary prevention and treatment.

**Methods:**

In this study, one hundred and thirty eight patients with cerebral ischemic events were enrolled; 100 patients with nonischemic cerebral diseases were enrolled as controls. Noninvasive ultrasound was used to measure the atherosclerotic plaques and intima-media thickness (IMT) of carotid and femoral artery.

**Results:**

Our results showed that patients in study group had higher incidence and severity of carotid and femoral plaques, and higher mean intima-media thickness (IMT) at both the carotid and femoral sites compared with that of controls (p < 0.01). Carotid atherosclerosis were highly prone to have instability plaques in study group(p < 0.001).

**Conclusions:**

This cross-sectional study showed that, the prevalence of carotid atherosclerosis and the unstable plaques were higher in cerebral ischemic patients.

**Keywords:**

Carotid artery; Atherosclerosis; Intima-media thickness; Cerebral ischemic stroke

## Introduction

Extracranial internal carotid artery stenosis (EICAS) accounts for approximately 25% of ischemic stroke. Previous literature has shown that even patients with asymptomatic EICAS require treatment. The soft and mixed plaques are unstable and may lead to rupture or atheroma, their presence might be at least equal to 50% stenosis [1]. Unstable plaques put patients at high risk for acute ischemic events. Intimamedia thickness (IMT) is positively correlated with atherosclerotic factors, the severity of arterial stenosis, and the resistance of the carotid artery [2]. In this study, we aimed to investigate the EICAS in the cerebral ischemic stroke in a Chinese population.

## Materials and Methods

### Patients

From April 2006 to December 2007, 138 patients (95 male, 43 female, mean age 63.76 ± 10.79 years) admitted for cerebral ischemic diseases as transient ischemic attack (TIA) or cerebral infarction were enrolled in this study, all patients were diagnosed according to the NINDS criteria (the National Institute of Neurological Disorder and Stroke). Patients were excluded from the study if they had a cardiac valvular or rhythm disorder which was likely to be associated with cardiogenic embolism.

Another 100 patients with age-, sex-matched (61 male, 39 female, mean age 61.41±11.53 years), were enrolled for controls, they were diagnosed as major depression, headache of tension type, Meniere's disease, and encephalitis.

### Imaging examination

All patients were performed brain CT scan and/or MRI at admission. Personal data such as medical history, family history, body mass index (BMI) were recorded. At the day of test, fasting blood were collected for determination of glucose, lipids profile, renal function (Beckman CX-9 Automatic Biochemical Analyzer).

If patients' condition permitted, the high-resolution ultrasound was used to measure common carotid, internal carotid, and femoral artery atherosclerotic plaques. The stability of plaque was determined according to the resound features and defined as soft, mixed and hard plaque. The IMT at 1- 1.5 cm of right common carotid and right femoral arterial distal from bifurcate were also determined. The total number of plaques in carotid and femoral separately in the same patient was counted and their thickness was added up to evaluate the severity of atherosclerosis. The grade of plaques were classified as follows: grade 0, non or the adding thickness < 1 mm; grade 1, the adding thickness 1.1 - 5mm; grade 2, 5.1-10 mm; grade 3, >10 mm [3].

### Statistics assay

Data were analyzed with SPSS-13. Values were expressed as mean ± SD. Comparisons were performed between the groups using Student's t tests, and Chi-Square analysis. Statistical significance was considered when P < 0.05.

## Results

### Baseline clinical characteristics of the patients

Patients in study group showed higher prevalence in hypertension history, high systolic blood pressure, diastolic blood pressure, fasting blood glucose, creatinine and urea; with low levels of high-density cholesterol compared with those of controls. There were no differences between sex, age, BMI, total cholesterol, triglyceride, low-density cholesterol, diabetes history between the two groups (Table 1).

**Table 1:** Baseline clinical features of the patients

	Patients	Controls	*P* value
Number	138	100	
Age	63.76 ± 10.79	61.41 ± 11.53	> 0.05
BMI	25.28 ± 4.61	25.39 ± 2.81	> 0.05
M/F	95/43	61/39	> 0.05
Hypertension	99 (71.74%)	55 (55%)	< 0.01
Systolic pressure	150.85 ± 24.64	140.27 ± 22.34	< 0.01
Diastolic pressure	87.46 ± 14.29	83.47 ± 12.07	< 0.05
T-Ch	4.85 ± 1.13	4.99 ± 1.0	> 0.05
TG	1.85 ± 2.16	1.65 ± 1.35	> 0.05
HDL	1.13 ± 0.34	1.26 ± 0.37	< 0.05
LDL	2.93 ± 0.89	3.07 ± 0.90	> 0.05
Urea	5.50 ± 2.05	4.71 ± 1.14	< 0.01
Creatinine	99.61 ± 22.15	88.13 ± 19.27	< 0.001
Diabetes history	33 (23.91%)	15 (15%)	> 0.05
Glucose	5.78 ± 2.08	5.07 ± 1.74	< 0.05

IMT: intima-media thickness; BMI: body mass index; M: male; F: female; T-Ch: total cholesterol ; TG: triglyceride; HDL: high-density lipoprotein cholesterol; LDL: low-density lipoprotein cholesterol.

### Atherosclerotic plaques and IMT in two groups

The grade of atherosclerotic plaques and femoral plaques were higher in patients group than in controls (p < 0.001, p < 0.05, respectively), and a higher mean IMT at both the carotid and femoral sites compared with controls (p < 0.01). There was significant difference between patients and controls in both left and right carotid plaques (p < 0.001, p < 0.01, respectively). Table 2.

**Table 2:** The atherosclerosis of cerebral ischemic patients

Group	N	Sum of carotid plaques	Left carotid plaques	Right carotid plaques	Right carotid IMT	Sum of femoral plauqes	Right femoral IMT
Controls	100	3.40 ± 4.50***	1.59 ± 2.46***	1.81 ± 2.71**	0.82 ± 0.24**	1.79 ± 3.64*	0.82 ± 0.20**
Patients	138	6.22 ± 5.87	3.21 ± 3.37	3.01 ± 3.14	0.91 ± 0.19	3.53 ± 3.90	0.96 ± 0.24

Student' t-test, * p < 0.05, ** p < 0.01, *** p < 0.001

### Comparison of the grade of plaques between the two groups

**Figure 1:**
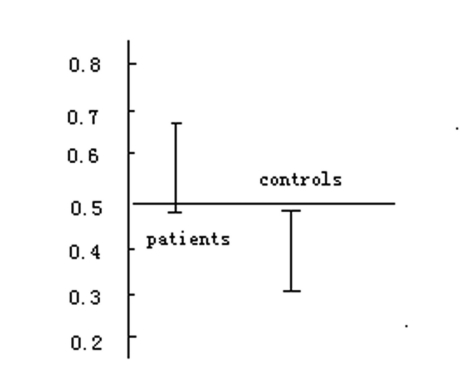
Mean Ridit values in study and control groups, p < 0.01.

Compared with that in the control group, the grade of plaques of carotid artery in the ischemic patients was higher (p < 0.01). The mean Ridit value of the ischemic patients was 0.577, 95% confidence interval (CI) was 0.6646-0.4894. The mean value of Ridit in the controls was 0.394, 95% CI is 0.4969-0.2911. There was significant difference between the two groups. (Table 3, Figure 1)

**Table 3:** The grade of the plaques in the patients and controls

Grade of plaques	Study	Control
0 (or < 1)	21 (15.22%)	42 (42%)
1 (1.1-5)	46 (33.33%)	34 (34%)
2 (5.1-10)	36 (26.09%)	13 (13%)
3 (> 10)	35 (25.36%)	11 (11%)

Ridit analysis, 95% confidence interval (CI) of the patients with ischemic events: 0.6646-0.4894, 95% CI of the controls: 0.4969-0.2911.

### Stable and unstable plaques in two groups

Carotid atherosclerosis was highly prevalent in study group comparing with controls. The prevalence of plaque of right common artery was 60.14% in study group, 46% in the controls (p < 0.05). The percentage of plaque of right internal artery was 24.64% in study group, 11% in controls (p < 0.01). Unstable plaque of right common carotid and right internal artery was significantly higher in study group than in the controls (84.34% vs 60.87%, p < 0.01; 82.35% vs 45.45%, p < 0.05; respectively). The prevalence of left common carotid in the study group was significantly higher than in the controls (71.01% vs 36%, p < 0.001). There was no significant difference of plaque prevalence in left internal carotid between the two groups (23.19% vs 13%, p > 0.05). The instability percentage of left common carotid was significantly higher in study group than in controls (61.59% vs 19%, p < 0.001). There was no significant difference of stability in left internal carotid between two groups. (Table 4, 5)

**Table 4:** Atherosclerotic plaques of right carotid in acute cerebral ischemic patients

Group	N	Right common carotid (n)			Right internal carotid (n)		
		with plaque	soft/mixed	hard	with plaque	soft/mixed	hard
Controls	100	46*	16/12**	18	11**	3/2*	6
Patients	138	83	42/28	13	34	15/13	6

Chi-square analysis, * p < 0.05, ** p < 0.01, controls vs patients.

**Table 5:** Atherosclerotic plaques of left carotid in acute cerebral ischemic patients

Group	N	Left common carotid (N)			Left internal carotid (N)		
		with plaque	soft/mixed	hard	with plaque	soft/mixed	hard
Controls	100	36***	8**/11***	17	13	3/5	5
Patients	138	98	50/35	13	32	11/13	8

Chi-square analysis, **p < 0.01, *** p < 0.001, patients versus control.

## Discussion

Cerebrovascular disease (CVD) or stroke is one of the first three leading causes of death in the past four decades in China. CVD is also the most important cause of disability in the elderly. CVD will still prevail in the future [5, 5]. The severity of atherosclerosis indicates the risks of the vascular events.

A prompt and accurate diagnosis of carotid artery disease is critical when planning a therapeutic strategy. Even in healthy people, the convenient surveillance of atherosclerosis is important in lifestyle modifications and pharmacological interventions. Physical examination is inaccurate in determining the presence and severity of carotid artery disease. Therefore, reliable imaging tests such as duplex ultrasonography which offer little risk to the patient are required [6]. The meta analysis showed the sensitivity of ultrasonography is approximately 94% and specificity is approximately 92% [7]. The Carotid intima-media thickness (CIMT) was found to correlate with transesophageal echocardiography (TEE) markers of aortic atherosclerosis, including complex aortic plaques, and combined cardiovascular sources of embolus (CSE). The optimal CIMT cutoff for detection of CSE on TEE was 0.78 mm [8]. The plaques are classified as stable and unstable according to the echo feature. Hypoechoic or heterogeneous hypoechoic plaques with an irregular surface or ulcerations have been considered soft/complex plaques at major risk of stroke; homogeneous hyperechoic plaques with smooth surface lesions have been considered simple or hard plaques at minor risk [9]. Study has shown that there is positive correlation between the peripheral artery atherosclerosis and carotid atherosclerosis [10], this implies that monitoring the plaque and thickness of the carotid or the femoral artery is important in preventing the stroke, especially in deciding the prophylactic methods to prevent the re-attack of the stroke.

In this study, the cerebral ischemic patients had higher prevalence in hypertension history, systolic and diastolic blood pressure, blood glucose, creatinine, urea, and had lower level of high-density cholesterol compared with controls. The results indicate the patients are prone to have metabolic disorders. Ishizaka has shown that chronic kidney dysfunction is related to the atherosclerosis [11]. We also showed the prevalence of the atherosclerosis in patients with cerebral ischemic disease was higher than in the controls. The unstable plaques that may lead to rupture, such as the soft or complex ones, are more often observed in the stroke patients. The IMT of common carotid or femoral artery were thicker than that in the controls. Our study further verified that atherosclerosis and its characteristic are the important risk factors in stroke onset.

Previous prospective cohort study showed an 11-15% decrease of stroke risk following every 10 mg/dl increase of HDL-C [12]. HDL-C protects against atherosclerosis through promoting cholesterol efflux. In addition, the antiinflammatory properties of HDL are important as well [13]. These findings suggest that the severer of the atherosclerosis, the harder in controlling risk factors. For those with vulnerable plaque or severe degree of stenosis ( ≥ 70%), we may turn to DSA (digital subtractive angiography) and carotid endarterectomy or stenting.

It is also important to monitor plaque evolution to prevent second stroke or asymptomatic carotid atherosclerosis changing to symptomatic CVD. Furthermore, patients with extracranial carotid atherosclerosis, either symptomatic or asymptomatic, demonstrate altered cerebral perfusion, leading to lacunar infarcts and periventricular and subcortical white matter lesion [14].
